# Developing medication independence: The experience of UK teenagers

**DOI:** 10.1002/bcp.70484

**Published:** 2026-02-13

**Authors:** Holly Hutchins, Charlotte King, Louis Bioletti, Tsegay Gebru, Daniel B. Hawcutt, Alder Hey Research Ambassadors

**Affiliations:** ^1^ Medical School University of Liverpool Liverpool UK; ^2^ Department of Women's and Children's Health University of Liverpool Liverpool UK; ^3^ Department of Health Data Science, Institute of Population Health University of Liverpool Liverpool UK; ^4^ NIHR Alder Hey Clinical Research Facility Alder Hey Children's Hospital Liverpool UK

**Keywords:** adolescence, independence, medication, school children

## Abstract

**Aims:**

There is a progression through childhood from being provided medications by caregivers to having to take responsibility for medications yourself, but little is known about when the transition of adolescents managing medicines begins. The aim of this study was to obtain a cross‐sectional sample of UK adolescents and when they become independent with medications.

**Methods:**

A prospective anonymous survey was conducted during the school academic year 2023/2024. Participating secondary schools were members of the Alder Hey Research Ambassador Scheme. This survey included questions to assess their demographics and usage of medications.

**Results:**

A total of 3164 responses were received from 18 schools and colleges. Among participants, 1749 (55.4%) were female, with a mean age of 15.3 years (range 13–18). In the last 6 months, 1414 (45%) participants had used prescription medications, and 1660 (53%) had used over‐the‐counter (OTC) medications. Overall, 937 (30%) participants reported using medications without parental knowledge, of which 67 (7.2%) were prescription medicines. Female adolescents were more likely than male adolescents for all ages to be using medicines without parental knowledge (*p* < 0.001). OTC medicines purchased were most commonly reported as analgesics (paracetamol and ibuprofen) and antihistamines.

**Conclusions:**

Young people acquire different aspects of medication independence throughout the teenage years and are varied among females and males. These data can inform the timing and content of educational information supplied to adolescents regarding medications and can be utilized by educational institutes in the United Kingdom, as well as healthcare and pharmaceutical industries to ensure their information is appropriate for different ages.

**What is already known about this subject?**

- There is no current guidance in the United Kingdom or the current UK data on when adolescents become independent in taking medications.
- Previous literature has focused on the prevalence of self‐medication among adolescents rather than age around independence with medication and age when adolescents start obtaining medications independently

**What this study adds?**

- Adolescents take responsibility for taking medications independently at younger ages than previously considered, as young as 13 years old.
- Over the counter medication is commonly obtained by adolescents under the age of 16. This highlights considerations for more stringent regulation in the United Kingdom.

## 1 INTRODUCTION

There are over 14 million children and young people (CYP) in the United Kingdom, just under 20% of the population.^1^ CYP utilize approximately 5% of all medications prescribed in healthcare.^2^ For babies and younger children, caregivers are responsible for all aspects of the child's health, including medications. Across childhood and adolescence, this responsibility starts to gradually shift from the parent to the young person. Legally, in the United Kingdom, there are several thresholds for the definition of ‘adult’, when formal responsibility is judged to have completely moved to the patient from the parent. It is not until 18 years old that a person is deemed to be fully an adult in relation to medical services; however, 16 and 17 year olds are assumed to have capacity, making decisions regarding their health and medication use.^3^

Access to medications is generally via one of two routes: over‐the‐counter (OTC) medications and prescription‐only‐medications (POM). In the United Kingdom, there is no legal age restriction placed on buying OTC medications, although most retailers restrict under‐16 s from making certain purchases.^4^ At 16 years old, adolescents can register with a General Practitioner (GP), access GP services online, order prescriptions and collect POMs.^5^ Under certain situations, under 16's can access POMs without parental input through sexual health and contraception services. In these services, 13–15‐year‐olds are given the same rights as over 16's unless there are concerns for wellbeing.^6^

For young people with long‐term conditions that require ongoing secondary care input into adulthood, transition clinics are an established practice, bridging the gap between paediatric and adult health care.^7^ Transition clinics have been designed to equip these adolescents with the information and resources required to manage their condition in adulthood. However, the majority of adolescents are not in this situation and are expected to develop the skills to manage their health independently. The UK national curriculum Personal Social Health and Economic (PSHE) syllabus requires some education around medicines, including information about what a prescription medication is, appropriate antibiotic usage and that medications can cause harms as well as benefits.^8^ Education around how to use health services in general, and prescriptions/OTC medications in particular, is not included.

Existing information in this area is limited. For young people undertaking ‘self‐medication’ (treating self‐recognized illness), the prevalence identified globally is highly variable (from 2% to 92%).^9^ In Germany, 25.2% of <17‐year‐olds had self‐medicated with 17% taking medication that was bought OTC. Self‐medication was associated with higher household income and maternal education level.^10^ In Brazil, 26.7% of 18‐year‐olds self‐medicated, and the practice increased with age and education level and was also impacted by familial behaviour with medication.^11^ Self‐medication presents a number of potential harms and could increase the burden of health services due to lack of knowledge regarding interactions, brand *vs*. drug names, overdosing and incorrect self‐diagnosis.

There are no current UK data on when adolescents start to take responsibility for their own medications. Previous UK data are almost 30 years old, only focused on two age bands (11–12‐ and 14–15‐year‐olds), and do not explore the impact of socio‐economic factors.^12^ There are no data on adolescence access to, and use of, OTC medications or ages that adolescents start using medications. This information is essential for both drug regulatory decisions as well as for schools, parents, and young people themselves to promote drug safety and knowledge.

Therefore, this project aimed to identify self‐reported medication behaviour among 13‐ to 18‐year‐old school aged pupils in the United Kingdom using an anonymous questionnaire.

## 2 METHODS

The project was part of the Alder Hey Research Ambassador (RA) scheme. This is a rolling UK‐based collaboration between secondary schools and a tertiary paediatric hospital in the North‐West of England that enables a range of secondary school pupils to contribute to relevant health issues through completion of anonymous questionnaires. In return, the scheme provides schools and individual ambassadors with research and work experience opportunities.^13^

The scheme involves secondary schools and colleges across the United Kingdom. Schools provided a named staff member and nominated two pupils as RAs. RAs were usually pupils in Year 10 or Year 12, ranging in age between 14 and 18 years. They were provided with training (outside of school hours) over Microsoft Teams and supported via email and online meetings. In return for their role within the scheme, RAs were prioritized for work experience and given appropriate recognition up to and including authorship in any published work (providing the required contributory criteria were met). Participating schools also received support for STEM (Science, Technology, Engineering and Mathematics) activities, careers events and PSHE (personal, health and social education) delivery in a bespoke manner. When the school was not located near Liverpool, a more local NIHR Clinical Research Facility (CRF) was contacted to support the work experience delivery. The project was registered via the Alder Hey Children's Hospital Audit department (reference 7028). The survey was anonymous, with no identifiable data collected, and in keeping with General Data Protection Regulation legislation (GDPR), only pupils aged 13 to 18 years old were eligible to complete it.^14^

Recruitment of schools, RAs and link staff commenced in September 2023 and continued until March 2024. RA training occurred throughout this period starting on 21 November 2023. RAs were provided with resources such as posters with QR codes (supplementary file) and presentations to support survey promotion and data collection. RAs were encouraged to promote the questionnaire through school assembly's, attending different year group classrooms and sending out the questionnaire electronically. The survey was hosted on Microsoft Teams for participants to complete. Informed consent was assumed via the participant's action of completing the survey and submitting it for analysis due to the survey being anonymous.

The questionnaire was open from November 2023 until May 2024 to provide sufficient time for RAs to disseminate the survey link. The survey consisted of 36 questions across 8 sections including scenarios. Questions covered if adolescents had long‐term health conditions or regular contact with health services, medication use in the previous 6 months, knowledge of prescription medication, age around taking medications and buying medications. RAs contributed to questionnaire development and the suitability of the questionnaire for the target age‐range (a full copy of which is available in the Supporting Information). The questionnaire was monitored for a drop off in participants, and schools were provided a warning prior to closure of the questionnaire.

RAs were provided with anonymized data and supported in undertaking data analysis to determine how adolescents at their schools were behaving. Anonymized participant data were extracted to Microsoft Excel at the closure of the questionnaire and disseminated to RAs. Free‐text answers underwent screening by a RA and a member of the study team; this involved responses to the question ‘What medications have you bought over the counter?’ in order to combine similar responses under generic medication terms. In addition, RAs were required to complete a school characteristics questionnaire to help facilitate analysis of socio‐economic factors that may impact the results. All data analysed by RAs were double checked and validated by the study team. Statistical analysis was performed via a two sample Z test or Chi‐square test. Two‐sample *Z* tests for proportions were used to compare cumulative proportions (percentages) of male and female adolescents for the following analysis: when do adolescents start taking medication, what age do adolescents access OTC medications and number and age of adolescents who reported ordering prescriptions over the internet. To examine the association between age and IMD decile for the following subsets: age at which medication is taken independently and access to OTC medications, a chi‐square test of independence was conducted. Observed counts of participants for each age *vs*. IMD category (IMD 1 or 10) were arranged in a 2 × 2 contingency table. Demographic characteristics and free text questions were presented as number of participants/medications and percentage.

The main outcomes were patterns of adolescent medication usage of both OTC and POM, ages at when adolescents start taking medications independently and which medications were obtained by adolescents.

### 2.1 Nomenclature of targets and ligands

Key protein targets and ligands in this article are hyperlinked to corresponding entries in http://www.guidetopharmacology.org and are permanently archived in the Concise Guide to PHARMACOLOGY 2021/22.^15^

## 3 RESULTS

**FIGURE 1:**
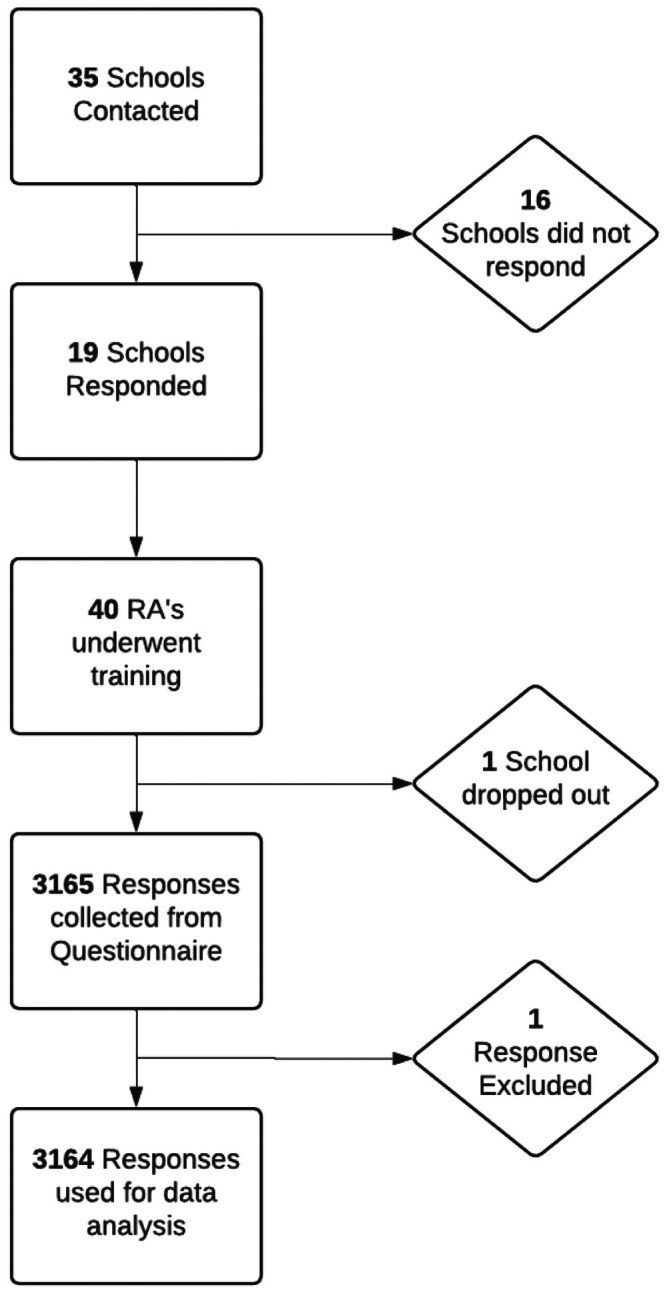
Flow diagram of school and research ambassador recruitment and participation.

Thirty‐five schools in total were contacted, with 22 proceeding to nominating link staff and RAs. Nineteen schools and colleges agreed to participate, and 18 of these returned completed surveys (and were therefore included in the results) as shown in Figure 1.

Summary data on the characteristics of participating schools are shown in Table 1 including IMD decile based on school postcode.

**TABLE 1:** The participating schools and their characteristics.

School name	Pupils in years 9–13	Type of school	Co‐educational or single sex?	Entry criteria?	Fee paying?	Number of responses to questionnaire	Postcode	IMD decile
Cardinal Newman College	4309	State	Co‐educational	Yes (GCSE's)	No	390	PR1 4HD	1
Formby High School	765	State	Co‐educational	No	No	215	L37 3HW	10
King David High School	542	Faith	Co‐educational	Yes (entrance exam)	No	372	L15 6WU	8
Lifesciences UTC	589	State	Co‐educational	Yes	No	152	L24 2UR	1
Loreto Grammar School	716	State	Single sex (girls)	Yes (entrance exam)	No	158	WA14 4AH	9
Merchant Taylors' Boys School	306	Private	Single sex (boys)	Yes (entrance exam)	Yes	69	L23 0QW	7
Merchant Taylors' Girls School	274	Private	Single sex (girls)	Yes (entrance exam)	Yes	86	L23 0QW	7
Ormskirk School	791	State	Co‐educational	No	No	142	L39 2AT	8
Riverside College and Cronton Sixth Form College	4600	State	Co‐educational	No	No	46	WA8 5WA	10
Saint Benedict Catholic Voluntary Academy	636	State	Co‐educational	No	No	485	DE22 1JD	10
St Philomena's Catholic High School for Girls	955	Faith	Single sex (girls)	No	No	97	SM5 3PS	9
The Belvedere Academy	740	State	Single sex (girls)	Yes (entrance exam)	No	159	L8 3TF	1
The Mosslands School	850	State	Single sex (boys)	No	No	36	CH45 8PJ	4
The Studio School	296	State	Co‐educational	No	No	63	L1 0BS	1
Ursuline High School	970	Faith	Single sex (girls)	Yes (religious affiliation)	No	33	SW20 8HA	10
Wimbledon College	850	Faith	Single sex (boys)	Yes (religious affiliation)	No	323	SW19 4NS	10
Wirral Grammar School for Boys	709	Grammar	Single sex (boys)	Yes (entrance exam)	No	143	CH63 3AQ	8
Wirral Grammar School for Girls	900	Grammar	Single sex (girls)	Yes (entrance exam)	No	194	CH63 3AF	8

*Note*: State refers to a nonselective government funded school, Faith refers to either a state‐funded or independently funded school that may have a religious process for admittance, Grammar refers to a state funded school which will have a selective entrance process usually through an exam; Private refers to a fee‐paying independent of government control school; and GCSE refers to General Certificate of Secondary Education.

A total of 3165 surveys were started; one was completely blank and therefore not included; as such, 3164 surveys with responses were returned. There was a mean age of 15.3 years, with all participants being between 13 and 18 years old. Pupils in Year 10 provided the most responses among the different year groups (*n* = 873); in the UK education system, Year 10 covers pupils ages 14–15 years old. The number of responses received from schools varied from 33 to 485 per school, with the median number of responses being 148 per school.

A full breakdown of the questions asked, and responses provided, is shown in the supplementary data section. The demographics and self‐reported health status of the participants answering the questionnaire are shown in Table 2.

**TABLE 2:** The demographics and self‐reported health status of the pupils completing the questionnaire.

Characteristic	*n*	%
Gender		
Female	1749	55.4%
Male	1372	43.4%
Prefer not to say	39	1.2%
Age		
13	336	10.4%
14	719	22.7%
15	717	22.6%
16	641	19.4%
17	553	17.5%
18	192	6.1%
Self‐rated health		
Excellent	915	28.9%
Good	1792	56.6%
Fair	384	12.1%
Poor	73	2.3%
Long‐term conditions		
Yes	739	23.1%
No	2425	76.6%
Do you regularly see a healthcare professional?		
1× a year	657	20.7%
1 to 4 × a year	705	22.3%
More than 4 times a year	271	8.6%
No	1531	48.4%

There were 71% of participants (*n* = 2252) who reported medication use in the previous 6 months. Of those who had used medication, 45% (1414) reported use of prescription medication and 53% (*n* = 1660) reported OTC medication use over the previous 6 months.

The majority of adolescents who partook in the survey (72%, *n* = 2279) reported they understood when and how to take medications, with 92% (*n* = 2915) aware of where medications were stored at home; these data are shown in the Supporting Information.

### 3.1 Medication taking without parental/carer knowledge

**FIGURE 2:**
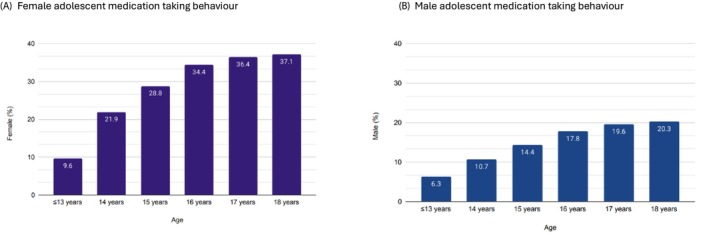
When do adolescents start taking medication without telling their parents/carers?

Table 3 shows the cumulative proportion of female and male adolescents who had begun using medication without telling their parents/carers at each age. The proportions for each age group are shown in Figure 2. It is consistently highlighted that females utilized medications throughout all ages compared to males without informing their parents (*p* < 0.001). Overall, the majority of participants had never taken medication without parental knowledge when compared to participants who had taken medications for both females and males (2324 (74%) participants *vs*. 834 (26%) participants). Adolescents who attended schools classified by postcode as IMD‐1 (most deprived) at age 16 were more likely to access medication without parental knowledge compared to those in IMD‐10 (least deprived), *p* < 0.05. This was not statistically significant for the other ages.

**TABLE 3:** Cumulative proportion of adolescents who began using medication without telling their parents/carers, by age and sex, with statistical comparisons at each age.

Age	Female	Male	Cum.Pct.Female	Cum.Pct.Male	CI lower	CI upper	*p*‐value
13	168	87	9.61	6.34	1.37	5.15	<0.0001
14	215	60	21.9	10.71	8.65	13.72	<0.0001
15	120	50	28.76	14.36	11.58	17.22	<0.0001
16	98	47	34.36	17.78	13.57	19.59	<0.0001
17	36	25	36.42	19.61	13.73	19.9	<0.0001
18	12	9	37.11	20.26	13.74	19.95	<0.0001

### 3.2 Access to OTC medications

**FIGURE 3:**
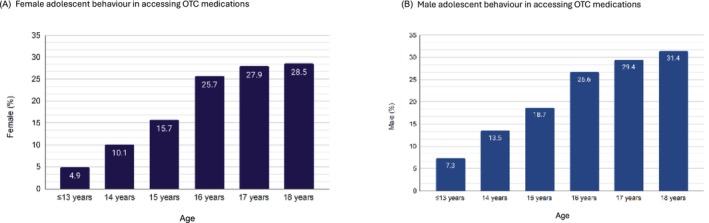
What age do adolescents access medications OTC?

Before 16 years old, OTC access did not differ meaningfully between female and male adolescents. From 16 years old onward, females were significantly more likely than males to access OTC medications, all *p* < 0.01; see Table 4. Cumulative access increased steadily with age, reaching 28.5% in females and 23.9% in males by age 18. The proportions of each age group are shown in Figure 3. When comparing IMD deciles for purchasing OTC medications, there was no significance between IMD 1 and 10 apart from in participants aged 16 years who were more likely to be in a IMD 1 compared to other ages, *p* < 0.05; this is shown in the Supporting Information.

**TABLE 4:** Cumulative proportion of adolescents who accessed over‐the‐counter (OTC) medications, by age and sex, with statistical comparisons at each age.

Age	Female	Male	Cum.Pct.Female	Cum.Pct.Male	CI lower	CI upper	*p*‐value
<13	86	76	4.92	5.54	−2.2	0.96	0.437
14	91	65	10.12	10.28	−2.3	1.98	0.886
15	95	54	15.55	14.21	−1.17	3.85	0.298
16	177	83	25.67	20.26	2.46	8.36	<0.01
17	39	29	27.9	22.38	2.48	8.57	<0.01
18	11	21	28.53	23.91	1.53	7.72	<0.01

For OTC medications, 82% (*n* = 2623) reported knowing that they could buy medicines from a shop or a pharmacy without a prescription. The majority had not purchased OTC medicines (73%, *n* = 2325), but 20% (*n* = 649) reported buying OTC medicines for themselves, and 14% (*n* = 428) had bought them for others among participants who responded to the survey.

The most reported medicines purchased OTC were paracetamol, ibuprofen and antihistamines (Table 5 shows the list of medications bought OTC and online).

**TABLE 5:** Medications reported as purchased over the counter (OTC) or over the internet.

Category	Responses	OTC	Internet
Analgesics	Paracetamol	16	7
	Ibuprofen	2	1
	Mefenamic acid	1	1
	‘Painkiller’	1	1
Antihistamines	Antihistamines	6	5
	Fexofenadine	1	1
	Cetirizine	1	1
Cold and flu	‘Cough medicine’	2	2
Acne	Benzoyl peroxide	3	2
	Benzoyl peroxide and clindamycin	1	
	Clindamycin and tretinoin	1	
	Tretinoin	1	
Contraceptives and hormonals	Norethisterone	4	5
	‘Birth control’	2	4
	Ethinylestradiol/levonorgestrel	1	

### 3.3 Ordering prescriptions over the internet

**FIGURE 4:**
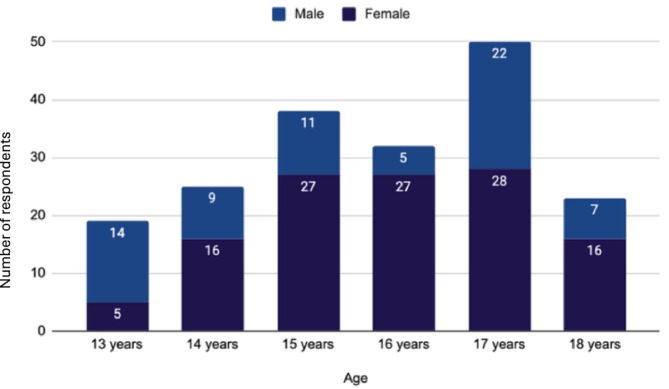
The number and age of adolescents who reported ordering prescriptions over the internet.

Participants highlighted the use of the internet in obtaining medications, with 4% (*n* = 120) having purchased medications over the internet. The proportion by age across males and females is shown in Figure 4. Across all ages, the cumulative proportion of adolescents ordering prescriptions online was low. At age 13, the cumulative proportion of male adolescents who had ordered prescriptions online was significantly higher than that of females (*p* = 0.009). No significant sex differences were observed at ages 14 or 15. From age 16 onwards, the cumulative proportion of female adolescents who had ordered prescriptions over the internet became higher than males, with statistically significant differences at ages 16 (*p* = 0.033) and 18 (*p* = 0.031) but not at age 17; see Table 6.

**TABLE 6:** Number, cumulative percentage, and age of adolescents who reported ordering prescriptions over the internet.

Age	Female	Male	Cum.Pct.Female	Cum.Pct.Male	CI lower	CI upper	*p*‐value
13	5	14	0.29	1.02	−1.32	−0.15	<0.05
14	16	9	1.2	1.68	−1.33	0.37	0.2632
15	27	11	2.74	2.48	−0.86	1.39	0.6443
16	27	5	4.29	2.84	0.15	2.74	<0.05
17	28	22	5.89	4.45	−0.11	2.99	0.0729
18	16	7	6.8	4.96	0.2	3.49	<0.05

Medications ordered over the internet included analgesia, oral contraception and norethisterone containing medications as well as antidepressants (Table 5).

## 4 DISCUSSION

This is the largest UK dataset on adolescent medication behaviour looking at independence with medication and use of both OTC and POM medications. The number of adolescents taking medications was similar to the national average of 40%–50%, and the number of young people with self‐reported long term health conditions was similar to the HSBC study from 2001–2022.^16^ In this study, the age of 16 was of specific interest as in the United Kingdom; it is often deemed as an age that adolescents assume capacity for healthcare decisions.^17^ For adolescents who purchased OTC medications, almost 60% were under 16 years old when they first started. In the United Kingdom, OTC medications can be bought in supermarkets and local shops not just in pharmacies. This allows adolescents to utilize self‐scanning of medications at automatic cash registers. This limits interaction between adolescents and pharmacy employees who may discuss benefits and risks of specific medications. Despite limitations retailers have around age‐restrictions for medications, these were not effective in preventing OTC purchasing in adolescents, as shown in 164 adolescents who first obtained OTC medications under 13 years of age.

The results highlighted how young people start to interact with medications independently at a lower age tharn possible considered by drug regulators and healthcare organizations. The adolescents responding were knowledgeable about medications; they understood how medications were stored at home, how to take medications and were aware of prescription medications and OTC medications. Males were more likely at age 13 to order prescriptions online (*p* = 0.009) compared to at 18 years when females were more likely (*p* = 0.031). This highlights the utilization of online resources to access medications among adolescents. This points to areas where educational institutions could focus on adolescents learning, such as the dangers of ordering medications off the internet without a formal health diagnosis.

Furthermore, we collected information regarding school characteristics allowing us to assign Index of multiple deprivation (IMD) rank to each school. There were four schools in the lowest quintile and five schools in the highest quintile. The results between the lowest and highest quintile were not statistically significant when comparing age of taking medications without parental knowledge and purchasing of OTC medications apart from those aged 16 years old in IMD 1 compared to IMD 10. Our evaluation is limited as school postcodes do not necessarily reflect adolescents home life and home postcodes; as the data were anonymous, we were unable to verify the data provided for participant postcodes. Prior studies have shown that adolescents living in households with higher incomes were more likely to purchase OTC medications which are the reverse of the results seen in this cohort.^10^, ^11^ One explanation is the demographical spread of schools included in the study; there were increased rates of private schools and grammar schools who participated in the RA scheme than is present in the general population. In the United Kingdom, private schools are fee‐paying institutions, independent of government funding; grammar schools are a type of state school but have a selective entry process in order for students to gain a place, whereas state schools are non‐selective. In future iterations of the RA scheme, efforts to expand the scheme to more geographical areas that represent the national picture should be made.

Limitations of the study include a disproportionate representation of private and grammar secondary schools compared to state schools in the United Kingdom. This variation is due to the scheme being established in the North‐West of England. This caused the majority of schools that were recruited to be from this region in England. The North‐West of England has a higher rate of grammar and single‐sex schools compared to other regions of England. Future work with the RA Scheme is to increase representation and inclusion for schools from all backgrounds across the United Kingdom to better reflect the national schooling picture. A further specific limitation revolved around the collection of information regarding acquisition of medications from the internet by adolescents. The questionnaire did not allow insight into how medications were obtained online; this limited analysis into whether adolescents referred to the utilization of online pharmacies, use of national health services phone application to order medication or used an unregulated online service. This should be reviewed if the questionnaire is repeated to address this issue.

## 5 CONCLUSION

Young people acquire different aspects of medication independence across the teenage years. These data can inform the timing and content of education about medications for young people and can be utilized by education, healthcare and pharmaceutical industries to ensure their information is appropriate for different ages.

## CONFLICT OF INTEREST STATEMENT

There were no competing interests.

## Supporting information


**Figure S1:** Posters provided to RAs regarding study.
**Table S1:** Full list of questionnaire and answers.
**Table S2:** Age and IMD decile comparison for when do adolescents start taking medication without telling their parents/carers?
**Table S3:** Age and IMD decile comparison for when adolescents access medications OTC.

## Data Availability

The data that support the findings of this study are available from the corresponding author upon reasonable request.
