# Supplementary material for: Developing medication independence: The experience of UK teenagers
Source: Br J Clin Pharmacol. 2026 Feb 13;92(7):2127–35. doi: 10.1002/bcp.70484 (PMC13304253; doi:10.1002/bcp.70484)
Supplement: Supplementary file 1 — Figure S1: Posters provided to RAs regarding study. Table S1: Full list of questionnaire and answers. Table S2: Age and IMD decile comparison for when do adolescents start taking medication without telling their parents/carers? Table S3: Age and IMD decile comparison for when adolescents access medications OTC. [file BCP-92-2127-s001.docx]

# Developing medication independence: the experience of UK teenagers (SUPPLEMENTARY DATA SECTION)

Holly Hutchins^1^, Charlotte King^2^, Louis Bioletti^1^, Tsegay Gebru^3^, Alder Hey Research Ambassadors*, Daniel B Hawcutt^2,4^

1: University of Liverpool Medical School, Liverpool, UK

2: Department of Women’s and Children’s Health, University of Liverpool, Liverpool, UK

3: Department of Health Data Science, Institute of Population Health, University of Liverpool, Liverpool, UK

4: NIHR Alder Hey Clinical Research Facility, Alder Hey Children’s Hospital, Liverpool, UK

*See Acknowledgments Section for the Alder Hey Research Ambassadors and Teacher Contacts.


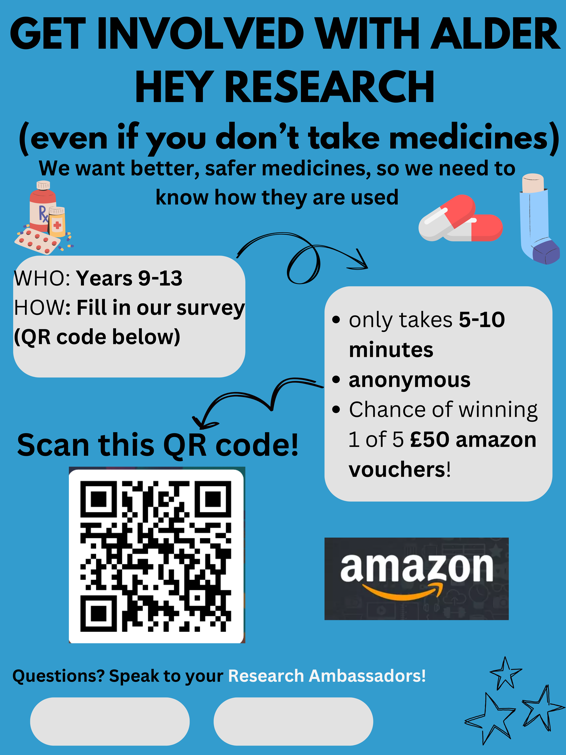

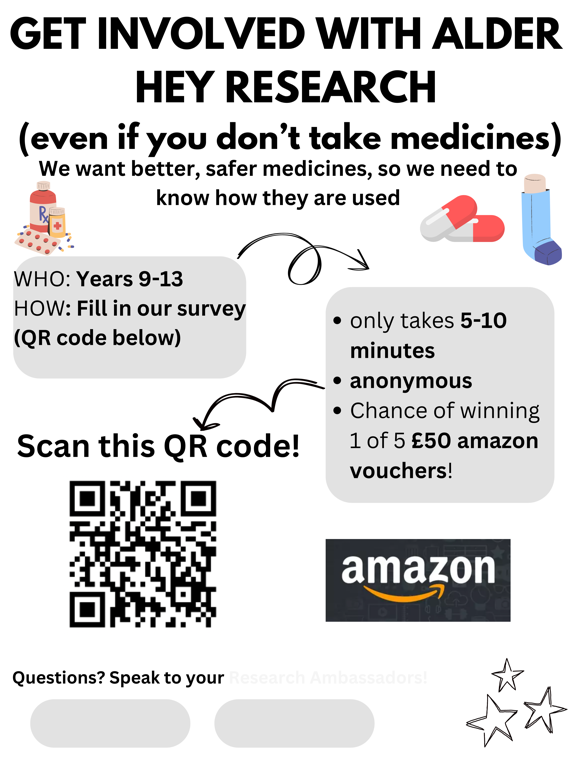


Figure S1 Posters provided for RAs regarding study

Table S1 – full list of questions and responses

| **Question** | | | | | **Age 13** | | **Age 14** | | **Age 15** | | **Age 16** | | **Age 17** | | **Age 18** |
| --- | --- | --- | --- | --- | --- | --- | --- | --- | --- | --- | --- | --- | --- | --- | --- |
| **Would you say your health is?** | | | | |  | |  | |  | |  | |  | |  |
| Excellent | | | | | 110 (33%) | | 234 (32%) | | 227 (32%) | | 168 (26%) | | 129 (23%) | | 46 (24%) |
| Good | | | | | 43 (13%) | | 70 (10%) | | 100 (14%) | | 66 (10%) | | 82 (15%) | | 22 (11%) |
| Fair | | | | | 176 (52%) | | 401 (56%) | | 376 (52%) | | 394 (62%) | | 330 (60%) | | 111 (58%) |
| Poor | | | | | 7 (2%) | | 14 (2%) | | 14 (2%) | | 13 (2%) | | 12 (2%) | | 13 (7%) |
|  | | | | |  | |  | |  | |  | |  | |  |
| **Do you have any long-term conditions e.g., asthma, epilepsy, diabetes, mental health?** |  | |  | | |  | | | |  | | | |  | |
| Yes | | | | | 261 (78%) | | 570 (79%) | | 559 (78%) | | 480 (75%) | | 413 (75%) | | 137 (71%) |
| No | | | | | 75 (22%) | | 149 (21%) | | 158 (22%) | | 161 (25%) | | 140 (25% | | 55 (29%) |
|  | | | | |  | |  | |  | |  | |  | |  |
| **Do you reguarly see a healthcare professional for a health condtion?** | | | | |  | |  | |  | |  | |  | |  |
| 1 times a year | | | | | 82 (24%) | | 158 (22%) | | 158 (22%) | | 133 (21%) | | 131 (24%) | | 37 (19%) |
| 1 to 4 times a year | | | | | 70 (21%) | | 174 (24%) | | 174 (24%) | | 143 (22%) | | 104 (19%) | | 39 (20%) |
| More than 4 times a year | | | | | 29 (9%) | | 50 (7%) | | 50 (7%) | | 58 (9%) | | 54 (10%) | | 21 (11%) |
| No | | | | | 155 (46%) | | 337 (47%) | | 337 (47%) | | 307 (48%) | | 264 (48%) | | 95 (49%) |
|  | | | | |  | |  | |  | |  | |  | |  |
| **Have you taken any medications in the last 6 months?** | | | | |  | |  | |  | |  | |  | |  |
| Yes | | | | | 233 (66%) | | 504 (70%) | | 515 (72%) | | 464 (72%) | | 395 (71%) | | 138 (72%) |
| No | | | | | 63 (19%) | | 150 (21%) | | 144 (20%) | | 141 (22%) | | 126 (23%) | | 47 (24%) |
| I don't know | | | | | 40 (12%) | | 65 (9%) | | 58 (8%) | | 36 (6%) | | 32 (6%) | | 7 (4%) |
|  | | | | |  | |  | |  | |  | |  | |  |
| **Have you taken a medication prescribed by a Doctor in the last 6 month?** | | | | |  | |  | |  | |  | |  | |  |
| Yes | | | | | 131 (39%) | | 286 (40%) | | 343 (48%) | | 300 (47%) | | 251 (45%) | | 99 (52%) |
| No | | | | | 167 (50%) | | 373 (52%) | | 337 (47%) | | 318 (50%) | | 283 (51%) | | 92 (48%) |
| I don't know | | | | | 38 (11%) | | 60 (8%) | | 37 (5%) | | 23 (3%) | | 19 (3%) | | 1 (0.5%) |
|  | | | | |  | |  | |  | |  | |  | |  |
| **Have you taken a medication from the shops or pharmacy (over the counter) that has not been prescribed in the last 6 months?** | | | | |  | |  | |  | |  | |  | |  |
| Yes | | | | | 145 (43%) | | 339 (47%) | | 357 (50%) | | 363 (57%) | | 330 (60%) | | 123 (64%) |
| No | | | | | 123 (37%) | | 266 (37%) | | 279 (39%) | | 221 (35%) | | 195 (35%) | | 62 (32%) |
| I don't know | | | | | 68 (20%) | | 114 (16%) | | 76 (11%) | | 55 (8%) | | 26 (5%) | | 7 (4%) |
|  | | | | |  | |  | |  | |  | |  | |  |
| **Do you know how to book a GP appointment?** | | | | |  | |  | |  | |  | |  | |  |
| Yes | | | | | 134 (40%) | | 339 (47%) | | 390 (54%) | | 418 (65%) | | 427 (77%) | | 157 (82%) |
| No | | | | | 202 (60%) | | 380 (53%) | | 327 (46%) | | 223 (35%) | | 126 (23%) | | 35 (18%) |
|  | | | | |  | |  | |  | |  | |  | |  |
| **If you have a medical issue and need to go to the GP, do you normally go with your parents/carers?** | | | | |  | |  | |  | |  | |  | |  |
| Never | | | | | 23 (7%) | | 50 (7%) | | 49 (7%) | | 25 (4%) | | 51 (9%) | | 30 (16%) |
| Rarely | | | | | 36 (11%) | | 46 (6%) | | 24 (3%) | | 32 (5%) | | 55 (10%) | | 30 (16%) |
| Sometimes | | | | | 28 (8%) | | 59 (8%) | | 57 (8%) | | 83 (13%) | | 86 (16%) | | 44 (23%) |
| Most of the time | | | | | 24 (7%) | | 70 (10%) | | 89 (12%) | | 120 (19%) | | 139 (25%) | | 39 (20%) |
| Always | | | | | 225 (67%) | | 494 (69%) | | 498 (69%) | | 381 (59%) | | 222 (40%) | | 49 (26%) |
|  | | | | |  | |  | |  | |  | |  | |  |
| **Have you ever ordered a prescription from the GP by yourself?** | | | | |  | |  | |  | |  | |  | |  |
| Yes | | | | | 21 (6%) | | 17 (2%) | | 36 (5%) | | 76 (12%) | | 130 (24%) | | 60 (31%) |
| No | | | | | 303 (90%) | | 685 (95%) | | 668 (93%) | | 556 (87%) | | 408 (74%) | | 129 (67%) |
| Don't know | | | | | 12 (4%) | | 17 (2%) | | 13 (2%) | | 9 (1%) | | 15 (2%) | | 3 (2%) |
|  | | | | |  | |  | |  | |  | |  | |  |
| **Do you take prescribed medicines that your parents/carers don't know about?** | | | | |  | |  | |  | |  | |  | |  |
| Yes | | | | | 14 (4%) | | 6 (1%) | | 10 (1%) | | 8 (1%) | | 19 (3%) | | 6 (3%) |
| No | | | | | 322 (96%) | | 713 (99%) | | 707 (99%) | | 633 (99%) | | 534 (97%) | | 186 (97%) |
|  | | | | |  | |  | |  | |  | |  | |  |
| **Have you ever collected a prescription for yourself from the GP?** | | | | |  | |  | |  | |  | |  | |  |
| Yes | | | | | 34 (10%) | | 77 (11%) | | 97 (14%) | | 147 (23%) | | 192 (35%) | | 93 (48%) |
| No | | | | | 277 (82%) | | 618 (86%) | | 601 (84%) | | 487 (76%) | | 347 (63%) | | 95 (49%) |
| Don't know | | | | | 25 (7%) | | 24 (3%) | | 19 (2%) | | 7 (1%) | | 14 (2%) | | 4 (2%) |
|  | | | | |  | |  | |  | |  | |  | |  |
| **Have you ever collected a prescription for someone else (family)?** | | | | |  | |  | |  | |  | |  | |  |
| Yes | | | | | 60 (18%) | | 124 (17%) | | 153 (21%) | | 167 (26%) | | 178 (32%) | | 66 (34%) |
| No | | | | | 253 (75%) | | 565 (79%) | | 543 (76%) | | 456 (71%) | | 361 (65%) | | 121 (63%) |
| Don't know | | | | | 23 (7%) | | 30 (4%) | | 21 (3%) | | 18 (3%) | | 14 (3%) | | 5 (3%) |
|  | | | | |  | |  | |  | |  | |  | |  |
| **Do you know where your medication is stored in your house?** | | | | |  | |  | |  | |  | |  | |  |
| Yes | | | | | 300 (89%) | | 652 (91%) | | 663 (92%) | | 598 (93%) | | 515 (93%) | | 181 (94%) |
| No | | | | | 22 (7%) | | 24 (3%) | | 21 (3%) | | 13 (2%) | | 14 (3%) | | 2 (1%) |
| N/A | | | | | 14 (4%) | | 43 (6%) | | 33 (5%) | | 30 (5%) | | 24 (4%) | | 9 (5%) |
|  | | | | |  | |  | |  | |  | |  | |  |
| **Are you on any long-term medication (taking longer than for 3 months)?** | | | | |  | |  | |  | |  | |  | |  |
| Yes | | | | | 67 (20%) | | 133 (18%) | | 168 (23%) | | 153 (24%) | | 147 (27%) | | 54 (28%) |
| No | | | | | 269 (80%) | | 586 (82%) | | 549 (77%) | | 488 (76%) | | 406 (73%) | | 138 (72%) |
|  | | | | |  | |  | |  | |  | |  | |  |
| **Do you know when and how to take your medications?** | | | | |  | |  | |  | |  | |  | |  |
| Yes | | | | | 262 (78%) | | 501 (70%) | | 529 (74%) | | 451 (70%) | | 390 (71%) | | 141 (73%) |
| No | | | | | 25 (7%) | | 32 (4%) | | 20 (3%) | | 20 (3%) | | 12 (2%) | | 3 (2%) |
| N/A | | | | | 49 (15%) | | 186 (26%) | | 168 (23%) | | 170 (27%) | | 151 (27%) | | 48 (25%) |
|  | | | | |  | |  | |  | |  | |  | |  |
| **Do your parents/carers supervise you when you take prescription medications?** | | | | |  | |  | |  | |  | |  | |  |
| Never | | | | | 33 (10%) | | 131 (18%) | | 174 (24%) | | 209 (33%) | | 213 (39%) | | 110 (57%) |
| Rarely | | | | | 53 (16%) | | 125 (17%) | | 124 (17%) | | 160 (25%) | | 153 (28%) | | 45 (23%) |
| Sometimes | | | | | 79 (24%) | | 147 (20%) | | 167 (23%) | | 136 (21%) | | 115 (21%) | | 24 (13%) |
| Most of the time | | | | | 89 (26%) | | 161 (22%) | | 126 (18%) | | 81 (13%) | | 44 (8%) | | 8 (4%) |
| Always | | | | | 82 (24%) | | 155 (22%) | | 126 (18%) | | 55 (8%) | | 28 (5%) | | 5 (3%) |
|  | | | | |  | |  | |  | |  | |  | |  |
| **Have you ever ordered a prescription over the internet? ( from an online pharmacist/doctor)** |  | |  | | |  | | | |  | | | |  | |
| Yes | | | | | 19 (6%) | | 27 (4%) | | 39 (5%) | | 32 (5%) | | 50 (9%) | | 23 (12%) |
| No | | | | | 278 (83%) | | 635 (88%) | | 630 (88%) | | 596 (93%) | | 476 (86%) | | 162 (84%) |
| I don't know | | | | | 39 (12%) | | 57 (8%) | | 48 (7%) | | 13 (2%) | | 27 (5%) | | 7 (4%) |
|  | | | | |  | |  | |  | |  | |  | |  |
| **Did you know you can buy medicines from a shop or a pharmacy (over-the-counter) without a prescription?** | |  | |  | | | |  | | | |  | | | |
| Yes | | | | | 234 (70%) | | 572 (80%) | | 577 (80%) | | 552 (86%) | | 501 (91%) | | 181 (94%) |
| No | | | | | 102 (30%) | | 147 (20%) | | 140 (20%) | | 89 (14%) | | 52 (9%) | | 11 (6%) |
|  | | | | |  | |  | |  | |  | |  | |  |
| **Have you ever bought a medicine from a shop or pharmacy (over-the-counter) for yourself?** |  | |  | | |  | | | |  | | | |  | |
| Yes | | | | | 43 (13%) | | 77 (11%) | | 99 (14%) | | 150 (23%) | | 193 (35%) | | 83 (43%) |
| No | | | | | 293 (87%) | | 642 (89%) | | 618 (86%) | | 491 (77%) | | 360 (65%) | | 109 (57%) |
|  | | | | |  | |  | |  | |  | |  | |  |
| **Have you ever ordered any medication from the internet?** | | | | |  | |  | |  | |  | |  | |  |
| Yes | | | | | 17 (5%) | | 18 (3%) | | 22 (3%) | | 20 (3%) | | 32 (6%) | | 10 (5%) |
| No | | | | | 298 (89%) | | 665 (92%) | | 664 (93%) | | 604 (94%) | | 508 (92%) | | 175 (91) |
| I don't know | | | | | 21 (6%) | | 36 (5%) | | 31 (4%) | | 17 (3%) | | 13 (2%) | | 7 (4%) |
|  | | | | |  | |  | |  | |  | |  | |  |
| **Have you ever bought a medicine from a shop or pharmacy (over the counter) for someone else?** |  | |  | | |  | | | |  | | | |  | |
| Yes | | | | | 30 (9%) | | 55 (8%) | | 67 (9%) | | 107 (7%) | | 127 (23%) | | 41 (21%) |
| No | | | | | 306 (91%) | | 664 (92%) | | 650 (91%) | | 534 (83%) | | 426 (77%) | | 151 (79%) |
|  | | | | |  | |  | |  | |  | |  | |  |
| **What age did you first buy over-the-counter medicine?** | | | | |  | |  | |  | |  | |  | |  |
| ≤13 years | | | | | 44 (13%) | | 55 (8%) | | 29 (4%) | | 20 (3%) | | 9 (2%) | | 6 (3%) |
| 14 years | | | | | 2 (<1%) | | 47 (6%) | | 60 (8%) | | 29 (5%) | | 17 (3%) | | 2 (1%) |
| 15 years | | | | | 0 (0%) | | 0 (0%) | | 34 (5%) | | 69 (11%) | | 37 (7%) | | 10 (5%) |
| 16 years | | | | | 0 (0%) | | 0 (0%) | | 2 (<1%) | | 80 (12%) | | 143 (26%) | | 37 (19%) |
| 17 years | | | | | 0 (0%) | | 0 (0%) | | 0 (0%) | | 0 (0%) | | 46 (8%) | | 22 (11%) |
| 18 years | | | | | 0 (0%) | | 3 (<1%) | | 1 (<1%) | | 0 (0%) | | 0 (0%) | | 30 (16%) |
| Never bought a medication | | | | | 290 (14%) | | 614 (85%) | | 591 (82%) | | 443 (69%) | | 301 (54%) | | 85 (44%) |
|  | | | | |  | |  | |  | |  | |  | |  |
| **Have you ever taken a medication that you have bought in a shop/pharmacy (over-the-counter)?** |  | |  | | |  | | | |  | | | |  | |
| Yes | | | | | 124 (27%) | | 257 (36%) | | 266 (37%) | | 293 (46%) | | 306 (55%) | | 120 (63%) |
| No | | | | | 212 (63%) | | 462 (64%) | | 451 (63%) | | 348 (54%) | | 247 (45%) | | 72 (37%) |
|  | | | | |  | |  | |  | |  | |  | |  |
| **How often have you taken over-the-counter medicine in the last six months?** | | | | |  | |  | |  | |  | |  | |  |
| Never | | | | | 189 (56%) | | 389 (54%) | | 349 (49%) | | 264 (41%) | | 227 (41%) | | 77 (40%) |
| Occasionally (at least once a month) | | | | | 109 (32%) | | 231 (32%) | | 269 (38%) | | 278 (43%) | | 249 (45%) | | 85 (44%) |
| Reguarly (more than once a month) | | | | | 38 (11%) | | 99 (14%) | | 99 (13%) | | 99 (15%) | | 77 (14%) | | 30 (16%) |
|  | | | | |  | |  | |  | |  | |  | |  |
| **Do you take over-the-counter medicines without your parents knowing?** | | | | |  | |  | |  | |  | |  | |  |
| Never | | | | | 300 (89%) | | 598 (83%) | | 529 (74%) | | 437 (68%) | | 338 (61%) | | 108 (56%) |
| Rarely (less than half the time) | | | | | 25 (7%) | | 62 (7%) | | 111 (15%) | | 87 (14%) | | 97 (17%) | | 40 (21%) |
| Sometimes (atleast half the time) | | | | | 7 (2%) | | 30 (4%) | | 46 (6%) | | 60 (9%) | | 65 (12%) | | 18 (9%) |
| Often (more than half the time) | | | | | 1 (<1%) | | 20 (3%) | | 21 (3%) | | 48 (7%) | | 42 (8%) | | 16 (8%) |
| Always (every time) | | | | | 3 (1%) | | 9 (1%) | | 10 (1%) | | 9 (1%) | | 11 (2%) | | 10 (5%) |
|  | | | | |  | |  | |  | |  | |  | |  |
| **What age did you start taking medication without telling your parent/carer?** | | | | |  | |  | |  | |  | |  | |  |
| ≤13 years | | | | | 53 (16%) | | 95 (13%) | | 55 (8%) | | 38 (6%) | | 12 (2%) | | 7 (4%) |
| 14 years | | | | | 3 (1%) | | 38 (5%) | | 105 (15%) | | 72 (11%) | | 52 (9%) | | 6 (3%) |
| 15 years | | | | | 0 (0%) | | 1 (<1%) | | 35 (5%) | | 70 (11%) | | 51 (9%) | | 14 (7%) |
| 16 years | | | | | 0 (0%) | | 1 (<1%) | | 1 (<1%) | | 46 (7%) | | 71 (13%) | | 26 (13%) |
| 17 years | | | | | 0 (0%) | | 0 (0%) | | 0 (0%) | | 1 (<1%) | | 40 (7%) | | 20 (10%) |
| 18 years | | | | | 2 (<1%) | | 0 (0%) | | 3 (<1%) | | 2 (<1%) | | 0 (0%) | | 16 (8%) |
| Never bought a medication without telling my parent/carer | | | | | 278 (83%) | | 584 (81%) | | 518 (72%) | | 412 (64%) | | 327 (59%) | | 103 (54%) |

Table S2: Age and IMD decile comparison for when do adolescents start taking medication without telling their parents/carers?

| **Age (years)** | **IMD 1** | **IMD 10** | **X^2^ test** | **P value** |
| --- | --- | --- | --- | --- |
| **<13** | 62 | 72 | 2.61 | 0.105 |
| **14** | 79 | 77 | 0.50 | 0.478 |
| **15** | 53 | 48 | 0.016 | 0.899 |
| **16** | 64 | 39 | 4.189 | 0.040 |
| **17** | 29 | 18 | 1.543 | 0.214 |
| **18** | 9 | 8 | 0.0001 | 0.993 |

Table S3: Age and IMD decile comparison for when adolescents access medications OTC

| **Age (years)** | **IMD 1** | **IMD 10** | **X^2^ Test** | **P value** |
| --- | --- | --- | --- | --- |
| **<13** | 47 | 48 | 0.571 | 0.450 |
| **14** | 48 | 56 | 2.399 | 0.121 |
| **15** | 55 | 48 | 0.0079 | 0.929 |
| **16** | 110 | 74 | 5.029 | 0.0249 |
| **17** | 29 | 21 | 0.549 | 0.458 |
| **18** | 11 | 19 | 3.394 | 0.065 |
